# Dynamic changes in intrathymic ILC populations during murine neonatal development

**DOI:** 10.1002/eji.201847511

**Published:** 2018-07-02

**Authors:** Rhys Jones, Emilie J. Cosway, Claire Willis, Andrea J. White, William E. Jenkinson, Hans J. Fehling, Graham Anderson, David R. Withers

**Affiliations:** ^1^ Institute of Immunology & Immunotherapy, College of Medical and Dental Sciences University of Birmingham Birmingham UK; ^2^ Institute of Immunology University of Ulm Ulm Germany

**Keywords:** Innate lymphoid cells, Lymphoid tissue, Neonate, RORγt, Thymus

## Abstract

Members of the innate lymphoid cell (ILC) family have been implicated in the development of thymic microenvironments and the recovery of this architecture after damage. However, a detailed characterization of this family in the thymus is lacking. To better understand the thymic ILC compartment, we have utilized multiple in vivo models including the fate mapping of inhibitor of DNA binding‐2 (Id2) expression and the use of Id2 reporter mice. Our data demonstrate that ILCs are more prominent immediately after birth, but were rapidly diluted as the T‐cell development program increased. As observed in the embryonic thymus, CCR6^+^NKp46^−^ lymphoid tissue inducer (LTi) cells were the main ILC3 population present, but numbers of these cells swiftly declined in the neonate and ILC3 were barely detectable in adult thymus. This loss of ILC3 means ILC2 are the dominant ILC population in the thymus. Thymic ILC2 were able to produce IL‐5 and IL‐13, were located within the medulla, and did not result from ILC3 plasticity. Furthermore, in WT mice, thymic ILC2 express little RANKL (receptor activator of nuclear factor kappa‐B ligand) arguing that functionally, these cells provide different signals to LTi cells in the thymus. Collectively, these data reveal a dynamic switch in the ILC populations of the thymus during neonatal development.

## Introduction

Innate lymphoid cells (ILC) have been described in many tissues 1, 2 and it seems likely that this family of cells is present essentially throughout the body. However, it is clear that the precise composition of these cells is often highly tissue specific and also developmentally regulated. Within the embryo, lymphoid tissue inducer (LTi) cells comprise the RORγt‐dependent group 3 ILC (ILC3) population and these cells appear, required for the establishment of secondary lymphoid tissues such as lymph nodes and Peyer's patches 3, through orchestrating recruitment of lymphocytes to the developing anlagen after LTi cell:stromal cell interactions 4. Notably, LTi cells were also identified within the embryonic thymus where they again interact with stroma to establish a lymphoid microenvironment, in this case providing receptor activator of nuclear factor kappa‐B ligand (RANKL) to developing medullary thymic epithelial cells (mTEC) 5. Thus in both primary and secondary lymphoid tissues, members of the ILC3 family play a key role in the generation of tissue microenvironments that support lymphocyte maturation and subsequent responses. Given the importance of lymphoid tissue microenvironments to normal function, recovery of such architecture is rapidly induced following damage and ILC3 have also been implicated in this process in both primary and secondary lymphoid tissue 6, 7. Recovery of splenic architecture after viral infection was impaired in *Rorc* ^−/−^ mice 6, while ILC3 have been identified as a key source of IL‐22 after radiation induced thymic damage 7. While the ILC compartment of secondary lymphoid tissues has been described in several studies 8, 9, characterization of ILC populations in the thymus has not been performed in detail, perhaps impeded by the phenotypic similarities between ILC and developing thymocytes. In addition to the descriptions of ILC3, ILC2 have also been identified in both mouse and human thymus 10, 11, 12, 13, although their location within the tissue and their function remain unclear. Furthermore, whether the ILC populations in the thymus change over time has not been addressed. Here we have undertaken a detailed characterization of thymic ILC from birth through to adulthood using several in vivo models to ensure discrimination of ILC from developing thymocytes. While we could clearly detect ILC3 with an LTi cell phenotype in embryonic and neonatal thymus, surprisingly we found that ILC3 rapidly declined in number after birth. In contrast, ILC2 gradually increased in the thymus over time. These cells were located within the medulla of the thymus, were functional in terms of their capacity to make IL5 and IL‐13, and lacked expression of RANKL, a molecule thought to be critical for LTi cell function in the developing thymus. Collectively, our data reveal that while ILC3 function to establish the thymic medulla, ILC2 become the dominant ILC population within the thymus after birth and likely support normal thymic function through type 2 cytokine release.

## Results

### ILC populations in the neonatal thymus

LTi cells were originally identified within the embryonic thymus more than 10 years ago 5. Since the discovery of LTi cells, it is now understood that these cells belong to the ILC family, with inclusion in this family dependent upon a clearly described phenotype and function 2. Given these advances, we wanted to reexamine the ILC populations within the embryonic thymus and asked whether any other ILC populations beyond LTi cells were present. To do this, we established fetal thymic organ cultures using embryonic day 16 thymi. After 7 days in culture, fetal thymic organ cultures were disaggregated and ILC populations were assessed by flow cytometry. ILC were defined as IL‐7Rα^+^Lin^−^CD8α^−^ intracellular CD3^−^ (CD3i^−^) cells with ILC2 and ILC3 identified on the basis of expression of the transcription factors GATA‐3 and RORγt (Supporting Information Fig. 1). In addition to a clear LTi cell population (RORγt^+^CCR6^+^NKp46^−^CD4^+/−^), a smaller, but distinct population of RORγt^−^GATA‐3^+^ ILC2 was evident, indicating that multiple ILC populations or at least their progenitors exist within the embryonic thymus after ex vivo culture condition.

**Figure 1:**
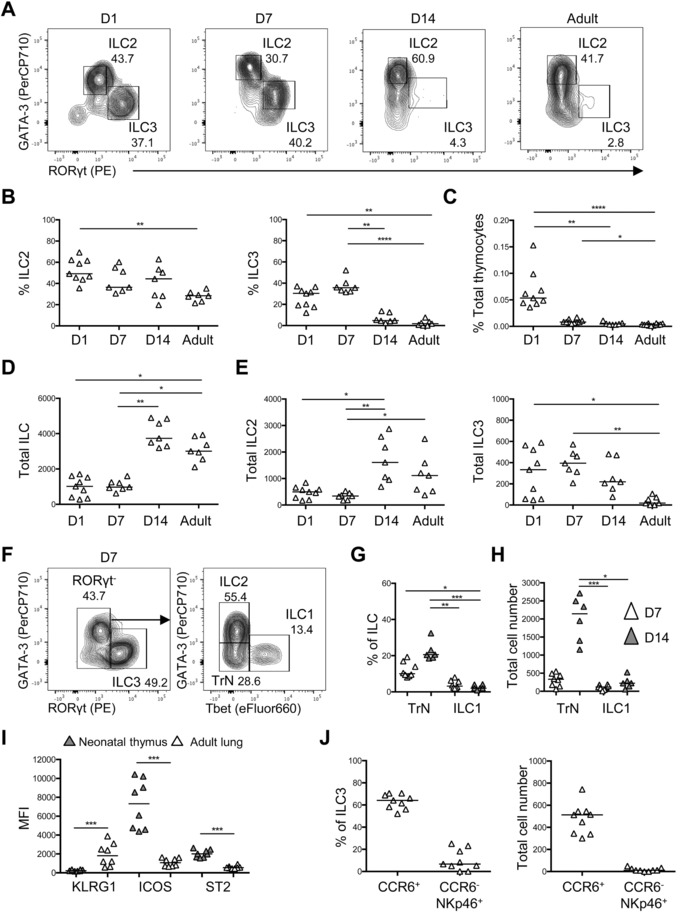
Characterization of thymic neonatal ILC populations. To assess the nature of ILC populations in the neonatal thymus, these cells were characterized at 1, 7, and 14 days postbirth and in adulthood (6–12 weeks) by flow cytometry. (A) Representative flow cytometry plots showing analysis of thymic ILC identified as CD8α^−^CD3i^−^IL‐7Rα^+^Lin^−^ (B220, CD3, CD5, CD11b, and CD11c) cells with ILC2 (GATA‐3^+^) and ILC3 (RORγt^+^) gated. (B) The proportion of ILC2 and ILC3 as a percentage of the total thymic ILC population. (C) The proportion of ILC as a percentage of total thymocytes. The total number of ILC (D) and the total number of ILC2 and ILC3 (E) in the thymus at different ages. (F) Representative flow cytometry plots showing analysis of thymic ILCs identified as CD8α^−^CD3i^−^IL‐7Rα^+^extended‐Lin^−^ (B220, CD3, CD5, CD11b, CD11c, CD19, CD49b, CD123, F4/80, FcεR1, Gr‐1, and Ter119) cells with ILC1 (Tbet^+^), ILC2 (GATA‐3^+^), ILC3 (RORγt^+^), and “triple negative” ILC (‘TrN’; GATA‐3^−^RORγt^−^Tbet^−^) gated in the neonatal thymus at 7 days postbirth. The proportion (G) and total number (H) of ‘TrN’ ILC and ILC1 in the neonatal thymus at 7and 14 days postbirth. (I) The median fluorescence intensity (MFI) of KLRG‐1, ICOS, and ST2 expression by ILC2 isolated from neonatal thymus and adult lung. (J) The proportion and total number of CCR6^+^NKp46^−^ and CCR6^−^NKp46^+^ ILC3 subsets in the neonatal thymus at 7 days postbirth. Mann–Whitney *U*‐test (comparing two samples) or a one‐way ANOVA (comparing three or more samples) was used for statistical analysis where *p < 0.05, **p < 0.01, ***p < 0.001, and ****p < 0.0001. In all graphs, the bar represents the median, *n* = 9 for day 1, *n* = 7 for day 7, *n* = 7 for day 14, and *n* = 7 for adulthood (apart from in F–H, where *n* = 8 for day 7 and *n* = 6 for day 14), with data (in panels B, C, D, E, G, H, I, and J) pooled from at least two independent experiments at each age. Full gating strategy for all flow cytometry data identifying thymic ILC populations is shown in Supporting Information Fig. 1A.

To understand whether these embryonic ILC populations were maintained after birth, we characterized ILC populations in the thymus at different ages (Fig. 1A). The neonatal thymus contained a mixture of ILC3 and ILC2 populations in similar proportions during the first week of life; however, by 2 weeks of age, the proportion of ILC that expressed RORγt had decreased sharply and very few ILC3 (<5% ILC population) were detected from this age onwards (Fig. 1A and B). The proportion of ILC among total thymocytes was highest at birth (∼0.05% of total thymocytes) but then rapidly declined, such that by 2 weeks of age, ILC formed a tiny (<0.001%) proportion of all thymocytes (Fig. 1C). Total numbers of ILC had actually increased between birth and 2 weeks of age (Fig. 1D), largely due to an increase in the total number of ILC2 (Fig. 1E). Total numbers of ILC3 had declined by 2 weeks of age and we could detect <50 ILC3 within an entire adult thymus (Fig. 1E). Having identified ILC2 and ILC3 populations, we additionally stained for expression of T‐bet to identify ILC1 in the thymus. At 7 and 14 days postbirth, a distinct ILC1 population was evident (Fig. 1F–H), demonstrating that the neonatal thymus contains all the ILC subsets. Notably, a population of ILC based on IL‐7Rα expression in the absence of expression of an extensive panel of lineage markers was also observed (Fig. 1F–H), consistent with a recent report identifying such cells in multiple tissues 14. In this report, prolonged enzymatic digestion was shown to increase the frequency of these cells prompting concerns as to their exact nature; however, here the thymus was disaggregated using only mechanical means. Combined these data indicate that after birth, the ILC3 compartment is gradually lost while a small ILC2 population is maintained through to adult hood. Phenotypically, the ILC2 in the neonatal thymus expressed little KLRG‐1, but did express ICOS and ST2, while the majority of the ILC3 compartment phenotypically resembled the CCR6^+^NKp46^−^ LTi cells observed in the embryo (Fig. 1I and J and Supporting Information Fig. 2).

### Identification of ILCs in the adult thymus

**Figure 2:**
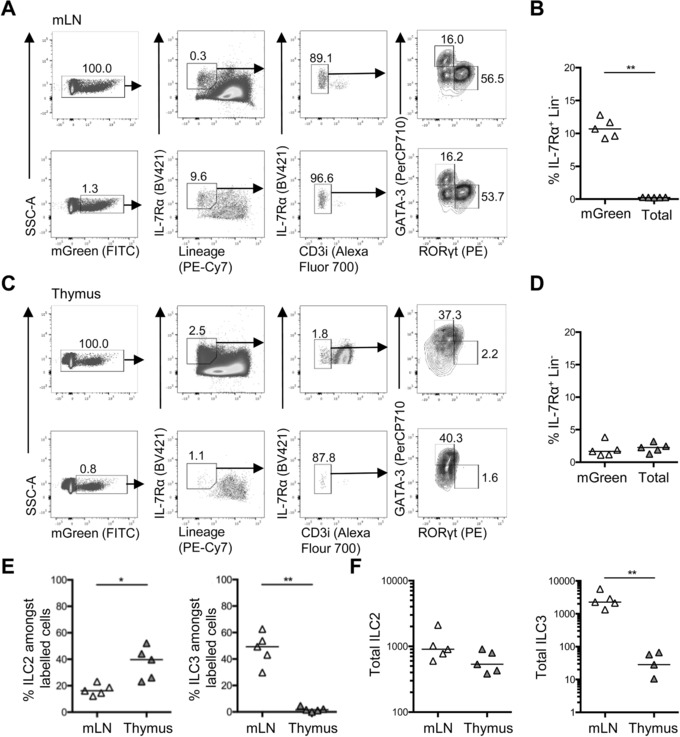
Analysis of thymic ILC populations through fate mapping Id2 expression. To aid identification of thymic ILC populations, Id2^creERT2^ × ROSA^mT/mG^ mice were used to fate map Id2 expression. Administration of tamoxifen by oral gavage on five consecutive days was performed and ILC populations in the thymus and mLN analyzed 3 days later by flow cytometry. (A) Full gating strategy for flow cytometry data showing analysis of IL‐7Rα^+^Lin^−^CD3i^−^ cells gated on all lymphocytes (upper panels) versus pregating on mGreen^+^ Id2 fate‐mapped cells (lower panels) among mLN cells. (B) The proportion of ILC in the mLN with and without gating on Id2 fate‐mapped (mGreen^+^) cells. (C) Representative flow cytometry plots showing analysis of IL‐7Rα^+^Lin^−^ CD3i^−^ cells from total cells (upper panels) versus pregating on mGreen^+^ Id2 fate‐mapped cells (lower panels) among thymus cells. (D) The proportion of ILC in the thymus with and without gating on Id2 fate‐mapped (mGreen^+^) cells. The proportion (E) and total number (F) of ILC2 and ILC3 in the mLN and thymus of Id2^creERT2^ × ROSA^mT/mG^ mice. Mann–Whitney *U*‐test was used for statistical analysis, where *p < 0.05 and **p < 0.01. In all graphs, the bar represents the median, *n* = 5, data (in panels B, D, E, and F) were pooled from two independent experiments.

ILC have a surface phenotype highly similar to T cells with the key exception of the TCR and associated signaling molecules. Given the variable expression of the TCR at different stages of thymocyte development, we considered that correct identification of ILC within adult thymus might be challenging using surface markers alone. Thus, we sought to confirm our initial data on ILC populations within the adult thymus and hypothesized that identification of cells that were fate mapped for inhibitor of DNA binding‐2 (Id2) expression would facilitate identification of ILC, since Id2 is a key transcription factor expressed by ILC progenitors 15, 16 but largely absent in, and not required by, developing thymocytes 17. To test this, we first assessed the ILC population detectable in the mesenteric lymph node (mLN) of Id2^creERT2^ × ROSA^mT/mG^ mice after tamoxifen administration by oral gavage (Fig. 2A). Comparing the ILC populations among total lymphocytes and those fate mapping for expression of Id2 (mGreen^+^), it was evident that prior gating on cells that were fate mapped for Id2 expression increased the proportion of IL‐7Rα^+^ Lin^−^ cells approximately 30‐fold (Fig. 2B), indicating that this approach could indeed facilitate identification of ILC. Importantly, the proportions of ILC2 and ILC3 identified through these gating strategies remained highly comparable. We then applied this gating approach to the identification of thymic ILC, which revealed a small fate‐mapped IL‐7Rα^+^Lin^−^ population in the thymus (Fig. 2C). While pregating on Id2‐fate‐mapped cells did not increase the proportion of ILC we identified, it did enable a more clear demarcation of a distinct IL‐7Rα^+^Lin^−^ population (Fig. 2C and D). Analysis of the adult thymus in Id2^creERT2^ × ROSA^mT/mG^ mice after tamoxifen showed that ILC3 were indeed a tiny proportion (<5%) of the ILC population in the thymus (Fig. 2D and E). To enumerate these ILC populations, the efficiency of ILC fate mapping in the mLN was first calculated and then used to generate total numbers in the thymus assuming comparable cre induction. Using this quantitation, we could again detect a median of <50 ILC3 per adult thymus, while the total number of ILC2 in the thymus was comparable to that detected in the mLN (Fig. 2F). An alternative approach administering tamoxifen for a longer period of time via the diet enabled an increased proportion of ILC to be fate mapped, however, the data was comparable in terms of the ILC subsets identified within the thymus (Supporting Information Fig. 3). Collectively, these experiments reveal that fate mapping Id2 expression does enhance our ability to identify ILC and within the adult thymus, the majority of ILC are not ILC3, but rather GATA‐3^+^ ILC2.

While assessing the cells in the thymus that fate mapped for Id2 expression, it was evident that the vast majority of these cells were not ILC. Using Id2^creERT2^ × ROSA26^tdRFP^ mice given tamoxifen by oral gavage for 5 days, we identified that the majority of thymic cells that were fate mapped (RFP^+^) expressed TCRβ^+^ (Supporting Information Fig. 4). Furthermore, it was observed that oral gavage with tamoxifen resulted in a substantial decline in thymocyte cellularity and double‐positive thymocytes were found to be particularly sensitive to administration of tamoxifen in this manner (Supporting Information Fig. 5). Analysis of ILC within the mLN revealed only a modest decrease in these cells indicating that the very low numbers of ILC detected in the thymus of Id2^creERT2^ × ROSA^mT/mG^ mice were not solely due to the effects of tamoxifen treatment (Supporting Information Fig. 4).

**Figure 3:**
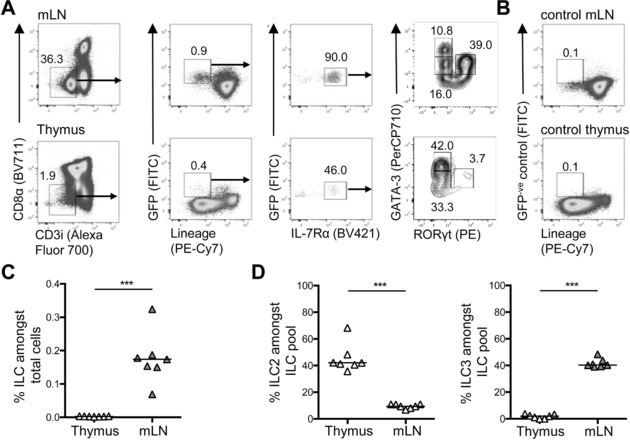
Analysis of thymic ILC in Id2‐eGFP reporter mice. To directly assess Id2 expressing ILC populations in the thymus and mLN, these tissues from Id2‐eGFP mice were analyzed by flow cytometry. (A) Full gating strategy for flow cytometry used to identify GATA3^+^ ILC2 and RORγt^+^ ILC3 among eGFP^+^ cells isolated from the mLN and thymus. (B) Gating controls for eGFP^+^ cells were based upon thymocytes from C57BL/6 WT mLN and thymus. (C) The percentage of eGFP^+^ ILC (CD8α^−^CD3i^−^IL‐7Rα^+^Lin^−^ cells) as a proportion of total cells isolated from thymus and mLN. (D) The percentage of eGFP^+^ ILC2 and ILC3 as a proportion of total ILC isolated from thymus and mLN. Mann–Whitney *U*‐test was used for statistical analysis, where ***p < 0.001, bars show medians, *n* = 7 for thymus and mLN. Data were pooled from two independent experiments.

To further analyze ILC populations using Id2 expression, but in the absence of tamoxifen administration, the ILC compartment in the mLN and thymus of Id2‐eGFP reporters was assessed (Fig. 3A). Cells isolated from WT mice (and thus eGFP^−^) were used to enable gating on eGFP^+^ populations (Fig. 3B). In the mLN, gating on eGFP^+^ cells enabled clear identification of ILC populations comparable to those described in the adult mLN 8. Consistent with the data from Id2^creERT2^ × ROSA^mT/mG^ mice, only a very small population of eGFP^+^Lin^−^ cells was detected in the thymus (Fig. 3A and C) and after gating on IL‐7Rα^+^ cells, ILC populations comparable to previous experiments were detected and arguing against an artefact of tamoxifen administration (Fig. 3A and D). Thus, using prior or current expression of Id2 to aid ILC identification, the data show that the adult thymus contains a small population of ILC in which ILC2 are far more numerous than ILC3.

### ILC2 are located in the thymic medulla

**Figure 4:**
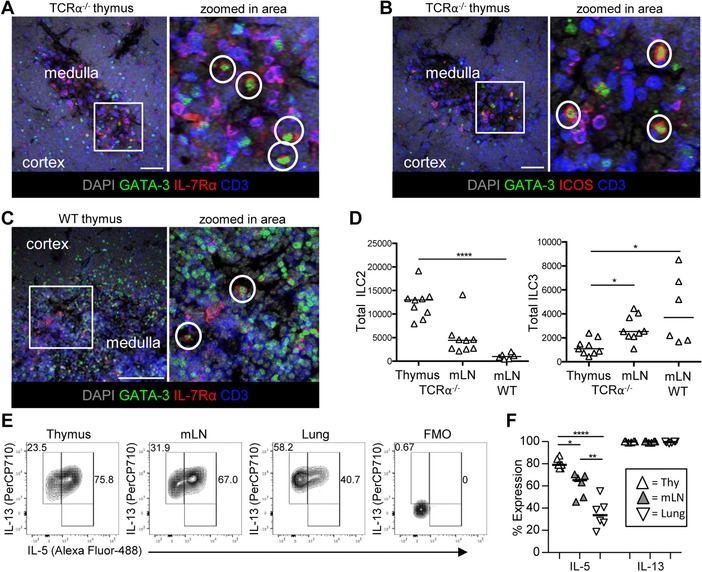
ILC2 reside within the medulla in the TCRα^−/−^ thymus. To investigate the location of ILC2 within the thymus, frozen sections of TCRα^−/−^ thymus were assessed for expression of (A) GATA‐3, CD3, and IL‐7Rα and (B) GATA‐3, CD3, and ICOS using immunofluorescence. Sections were counterstained with DAPI, data representative of tissue from three mice, putative ILC2 encircled in white. Scale bar: 50μm. (C) Sections of WT neonatal thymus assessed for expression of GATA‐3, CD3, and IL‐7Rα. Sections were counterstained with DAPI, data representative of tissue from three mice, scale bar represents putative ILC2 encircled in white. Scale bar: 100μm. (D) Total numbers of ILC2 and ILC3 isolated from TCRα^−/−^ thymus, TCRα^−/‐^ mLN, and WT mLN. (E) Representative flow cytometry plots showing expression of IL‐5 versus IL‐13 by ILC2 after ex vivo stimulation, alongside FMO control. Cells isolated from TCRα^−/−^ thymus, mLN, and lung were compared. (F) The proportion of ILC2 isolated from the thymus, mLN, or lung expressing IL‐13 and IL‐5 after ex vivo stimulation. Bars show medians, *n* = 9 for TCRα^−/−^ thymus and TCRα^−/−^ mLN, and *n* = 6 for WT mLN (C). *n* = 6 for thymus, mLN, and lung (D, E, and F). Mann–Whitney *U*‐test (comparing two samples) or a one‐way ANOVA (comparing three or more samples) was used for statistical analysis, where *p < 0.05, **p < 0.01, ***p < 0.001, and ****p < 0.0001. Data were pooled from two independent experiments. Full gating strategy for all the flow cytometry data used to identify ILC in TCRα^−/−^ mice shown in Supporting Information Fig. 6A.

Having characterized the ILC populations of the thymus, we wanted to better understand the location of these cells and what signals within the thymic microenvironment impacted on their development and persistence. Analysis of ILC in sections of lymphoid tissue is challenging given the abundance of T cells, which likely surround ILC populations and hinder identification of non‐T cells. Thus, we reasoned that it would be easier to identify ILC populations in the thymus using TCRα^−/−^ mice and here we could clearly identify putative ILC2 (GATA‐3^+^CD3^−^IL‐7Rα^+^) scattered within the medulla using immunofluorescence (Fig. 4A). Analysis of ICOS expression identified a similar population (this time GATA‐3^+^CD3^−^ICOS^+^ cells) within the thymic medulla (Fig. 4B) providing further evidence that these cells were indeed ILC2. Staining of 7 day old WT thymus for expression of GATA‐3^+^, CD3^−^ and IL‐7Rα^+^ again identified putative ILC2 in the medulla indicating that this location was true of both TCRα^−/−^ and WT thymi (Fig. 4C). Flow cytometric analysis of the ILC populations within the TCRα^−/−^ thymus confirmed that the majority of ILC belonged to the ILC2 group (Fig. 4D). Thus, normal medullary development, which is impaired in the absence of single positive CD4 T cells, is not required for the predominance of ILC2 among ILC populations in the thymus. To confirm that thymic ILC2 were able to express the signature cytokines associated with ILC2 function, ex vivo stimulations were performed and expression of both IL‐5 and IL‐13 detected (Fig. 4E and F). As observed in the mLN previously 8, ILC numbers were elevated within the TCRα^−/−^ thymus, but the vast majority remained ILC2 and the surface phenotype was consistent with that observed in the neonate (Supporting Information Fig. 6). The few thymic ILC that expressed RORγt also expressed CCR6 and were a mixture of CD4^+/−^ cells, consistent with the LTi‐like subset.

### ILC2 expansion in the thymus is not due to ILC3 plasticity

**Figure 5:**
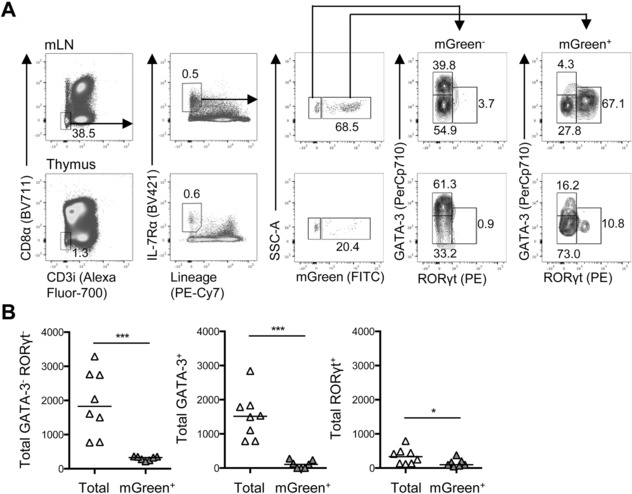
ILC3 plasticity does not account for increase in thymic ILC populations post birth. To test whether the loss of thymic ILC3 after birth could be attributed to differentiation to another ILC type, RORγt expression was fate mapped using *Rorc* ^cre^ × ROSA^mT/mG^ mice and cells analyzed by flow cytometry. (A) Full gating strategy for flow cytometry data identifying mGreen^+^ (fate mapped) and mGreen^−^ populations among ILC (CD8^−^CD3i^−^IL‐7Rα^+^Lin^−^) in the mLN and thymus. (B) Enumeration of thymic ILC showing putative ILC1 (GATA3^−^RORγt^−^), ILC2 (GATA3^+^), and ILC3 (RORγt^+^) comparing numbers of each population among total ILC versus mGreen^+^ ILC. Mann–Whitney *U*‐test was used for statistical analysis, where *p < 0.05. Bars show medians, *n* = 8, pooled from two independent experiments.

Recent studies have identified plasticity among ILC populations, particularly within the ILC3 subset, where in vivo fate mapping studies have revealed that some ILC3 lose expression of RORγt to become “ex‐ILC3,” a population very similar to IFNγ^+^Tbet^+^ ILC1 18. Thus, it was conceivable that the loss of ILC3 in the thymus reflected plasticity during neonatal development with the RORγt‐expressing ILC population switching to another fate. To test whether ILC3 populations in the thymus show evidence of plasticity, we fate mapped RORγt expression in thymic ILC using Rorc^cre^ × ROSA^mT/mG^ mice and looked at mGreen expression in the thymus and mLN. While the majority of ILC in the mLN were mGreen^+^, reflecting the large number of ILC3 in this tissue 8, only a minority of ILC (∼20%) in the thymus were mGreen^+^ (Fig. 5A). Analysis of RORγt expression at the protein level using intracellular flow cytometry revealed a clear “ex‐ILC3” population in both the mLN and the thymus (mGreen^+^ but RORγt^−^). Enumeration of the number of total versus mGreen^+^ ILC populations made it clear that the vast majority of thymic ILC showed no evidence of previous expression at the *Rorc* locus, arguing against ILC3 plasticity accounting for the loss of ILC3 and the increase in ILC2 (Fig. 5B).

### ILC expression of RANKL in the neonatal thymus

**Figure 6:**
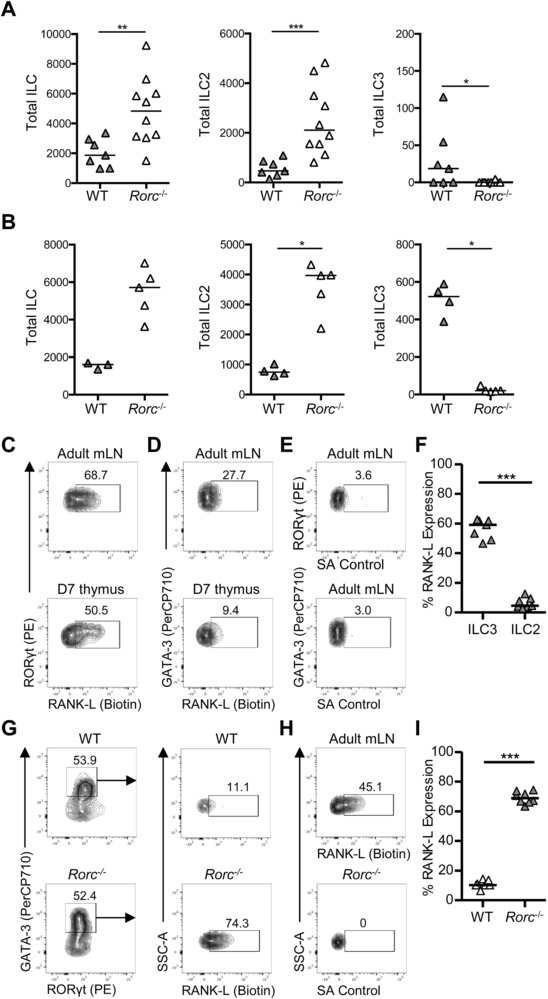
Neonatal ILC3, but not ILC2 express RANKL. To investigate the effect of *Rorc^−/−^* deletion on thymic ILC populations, ILC2 and ILC3 were enumerated in WT and *Rorc* ^−/−^ thymi. Numbers of ILC (IL‐7Rα^+^Lin^−^CD8^−^CD3i^−^), ILC2 (GATA‐3^+^), and ILC3 (RORγt^+^) detected in adult (A) and 7‐day‐old neonatal (B) thymus. *n* = 7 adult WT, 10 adult *Rorc* ^−/−^, four neonatal WT, and five neonatal *Rorc* ^−/−^. Expression of RANKL versus RORγt (C) and GATA‐3 (D) by ILC (IL‐7Rα^+^Lin^−^CD8^−^CD3i^−^) from adult mLN (upper panel) and neonatal thymus (lower panel) assessed by flow cytometry. (E) Staining control of SA‐PECy7 only with ILC2 and ILC3 from adult mLN. (F) The percentage of ILC2 and ILC3 expressing RANKL in the neonatal thymus, *n* = 7. (G) Expression of RANKL by GATA‐3^+^ ILC2 from WT (upper panels) and *Rorc* ^−/−^ (lower panels) 14‐day‐old neonatal thymus. (H) Controls for RANKL showing positive staining of RANKL by ILC from the mLN and the negative control (SA‐PECy7 only) for neonatal thymus. (I) The percentage of ILC2 isolated from WT and *Rorc* ^−/−^ neonatal thymus that express RANKL, *n* = 8. Mann–Whitney *U*‐test was used for statistical analysis, where **p < 0.01, bars show medians. Data were pooled from two (A, C, D, and F) and one (B and G) independent experiments. Full gating strategy for all flow cytometry identifying thymic ILC populations is shown in Supporting Information Fig. 1A.

The data described here reveal that the ILC compartment of the thymus changes after birth with a substantial decline in ILC3 number and increased numbers of ILC2. Given the increase in ILC2 numbers that coincided with the loss of ILC3 and the evidence that ILC3 exist in the medulla 5, we postulated that ILC2 numbers might be enhanced in the absence of ILC3. To test this, we analyzed ILC populations in the thymus of WT and *Rorc* ^−/−^ mice. Within the adult thymus, ILC2 numbers were significantly enhanced (>2‐fold) compared to WT controls (Fig. 6A). This increase in number was also evident in the neonate (Fig. 6B). While these data are consistent with competition for a medullary niche, the perturbations to T‐cell development in *Rorc* ^−/−^ mice mean that ILC intrinsic versus T‐cell‐dependent effects cannot be distinguished. The ILC3 population in the thymus prebirth has been implicated in mTEC maturation through provision of RANKL. While after birth T cells are considered likely cellular providers of RANKL to mTEC, we assessed RANKL expression among neonatal thymic ILC3 and ILC2 to test whether ILC2 might provide RANKL after the decline in ILC3 numbers. Using adult mLN cells as a positive control for RANKL expression, robust RANKL expression was detected on neonatal ILC3, however there was very little detected on ILC2 (Fig. 6C–F). These data indicate that the expanding ILC2 population in the thymus do not take over the provision of RANKL to mTEC as ILC3 decline. Surprisingly, however, significantly enhanced expression of RANKL was detected on ILC2 in the neonatal thymi of *Rorc* ^−/−^ mice, suggesting that ILC2 can express RANKL when the thymic microenvironment is perturbed.

## Discussion

Here we provide the first detailed characterization of ILC in the thymus, utilizing several in vivo models to facilitate accurate identification of these cells. Building on the earlier observation that LTi cells populate the embryonic thymus and orchestrate mTEC maturation through RANK:RANKL interactions, we demonstrate that after birth, LTi cells decline during neonatal development while ILC2 increase in number and become the main ILC population present within the thymus. We provide evidence that thymic ILC2 are able to produce the signature cytokines IL‐5 and IL‐13 and reside in the medulla where they may compete for an environmental niche with ILC3. Finally, we show that thymic ILC2 do not express RANKL under normal conditions, and thus appear to have distinct functions to thymic LTi cells during embryonic development.

ILC have been characterized within most tissues; however, to date a clear description of the thymic ILC compartment has been lacking. Discrimination between ILC and developing thymocytes where TCR expression is reduced is technically challenging, but this problem was resolved through analyzing expression of Id2 given its fundamental role in ILC development and redundant role in αβ TCR^+^ T cell development 17. Using both fate mapping and reporting of Id2 expression, our data clearly identify thymic ILC, but reveal that these cells account for less than 0.05% of the hematopoietic compartment of the thymus after birth and less than 0.001% in the adult thymus. Thus, ILC are certainly a rare population in the thymus. Functionally, LTi cells in the embryonic thymus provide the RANKL signals required for mTEC maturation. It is now evident that RANKL can be provided by other cells in the developing embryonic thymus 19 and after birth, single‐positive thymocytes alongside other T cell populations contribute to a complex network of signals governing mTEC development 20, 21, 22. Thus, the loss of LTi cells in the neonatal thymus is consistent with changes in the cellular interactions that govern mTEC maturation. Our data would argue that while ILC2 reside within the medulla, they are unlikely to contribute to mTEC maturation via RANKL provision. Thus, the role for ILC in establishing thymic microenvironments is restricted to early embryonic development.

What then do ILC2 contribute to thymic function? Based on the array of studies in other tissues, it is reasonable to hypothesize that ILC2 in the thymus are a local source of type 2 cytokines. Type 2 cytokines from iNKT cells were recently shown to control thymic emigration of conventional thymocytes 23, and it is possible that ILC2 may also contribute to this cytokine milieu. Whether ILC2 have unique functions within the thymus, or redundancy with other innate populations also able to produce similar cytokines such as iNKT cells requires further study, including the specific deletion of ILC2 24. Since mice lacking ILC3 had increased numbers of ILC2 within the thymus, there may be intrathymic competition among ILC for residency within the tissue. However, it is possible that the impaired T cell development caused by *Rorc* deficiency may also contribute to enhanced ILC2 numbers, particularly since a similar increase in thymic ILC2 was observed in the TCRα^−/−^ thymus, where numbers of single‐positive thymocytes are obviously greatly reduced.

In summary, this study provides new insight into the changes that occur in ILC populations within the thymus during neonatal development. The challenge remains to determine unique in vivo roles for these cells, particularly within nonmucosal tissues.

## Materials and methods

### Mice

Mice were obtained from the University of Birmingham Biomedical Services Unit (BMSU) and were maintained in accordance with Home Office regulations. All mice were on a C57BL/6 background and strains included: WT, Id2^creERT2^ 25 × ROSA^mT/mG^ 26, Id2^creERT2^ × ROSA26^RFP^ 27, Id2‐eGFP 28, *Rorc* ^−/‐^ 29, *Rorc* ^cre^ 30 × ROSA^mT/mG^, and TCRα^–/–^ 31. Neonatal mice were culled at day 1, 7, and 14 postbirth, and adult mice were culled between 6 and 12 weeks of age.

### In vivo procedures

Tamoxifen was administered to Id2^CreERT2^ × ROSA^mT/mG^ mice either by oral gavage (20mg/mL) once a day for five consecutive days and mice culled 3 days later or within diet for 3‐week duration. Tamoxifen was administered to Id2^creERT2^ × ROSA26^RFP^ mice by oral gavage (20mg/mL) once a day for three consecutive days and mice analyzed the following day.

### Cell preparation

Thymus tissue was mechanically disaggregated between two glass slides. mLNs were cleaned of all fat, teased apart using fine forceps, and DNase I (0.025mg/mL, Roche Diagnostics) and collagenase dispase (0.25mg/mL, Roche Life Sciences) were used to digest tissue in Roswell Park Memorial Institute (RPMI). All cell suspensions were passed through a 70‐μm nylon strainer (Falcon®, Fisher scientific) using a syringe plunger.

### Fetal thymic organ cultures

The embryonic sack containing stage E16 mouse embryos was removed from a pregnant mouse following cervical dislocation. Each embryo was decapitated and cut along the breastbone to expose two thymic lobes, which were dissected under a light microscope in sterile conditions. Fetal thymic organ cultures were set up in optimal conditions as previously described by Jenkinson and Anderson 32. Thymic lobes were placed on an Isopore™ Membrane Filter (0.8μm, Millipore) and positioned on top of a 1cm^2^ artiwrap sponge within a small petri dish containing 2 mL DMEM (Sigma–Aldrich). Each petri dish was encapsulated within a sealed container containing 10 mL of H_2_O and incubated for 7 days at 37°C/5% CO_2_. Single cell suspension from thymus tissue was prepared as previously described.

### In vitro culture for RANKL expression

Thymocytes prepared from neonatal thymi were cultured in 1 mL of culture media (RPMI, 10% FBS, L‐Glutamine, 100 IU/mL penicillin and 100 μg/mL streptomycin) overnight at 37°C/5% CO_2_. Wells were set up in duplicate for each sample with each well containing ∼6 million cells. Duplicate wells were pooled following culture to provide a sufficient number of ILC for analysis.

### Flow cytometry

Cell surface staining was performed at 4°C for 30 min in FACS buffer (10% FBS, 2.5mM EDTA in PBS). ILC were identified among CD8α^−^ (clone 5.3–6.7, Biolegend), CD3i^−^ (clone 17A2, Biolegend), IL‐7Rα^+^ (clone A7R34, Biolegend), B220^−^ (clone RA3‐6B2, eBioscience), CD11b^−^ (clone M1/70, eBioscience), CD11c^−^ (clone N418, eBioscience), CD3^−^ (clone 145‐2C11, eBioscience), and CD5^−^ (clone 53–7.3, eBioscience). Thymic iNKT cells were identified as mCD1d/PBS57^+^ and TCRβ^+^ (clone H57‐597, eBioscience). Intracellular cell staining was performed at room temperature for 1 h in permeabilization buffer (eBioscience). ILC2 and ILC3 subsets were identified using Abs against GATA3^+^ (clone TWAJ, eBioscience) and RORγt^+^ (clone AFKJS‐9, eBioscience). Staining for RANKL expression was performed in two steps using biotinylated RANKL (clone: IK22/5, eBioscience) and streptavidin‐PECy7 (Molecular Probes) at 4°C for 30 min in FACS buffer. Samples included Spherotech Accucount blank particles to enable calculation of cell frequency and were acquired using a Fortessa (BD). Samples were analyzed using FlowJo (FlowJo, LLC). Full gating strategy for all flow cytometry data identifying thymic ILC populations is shown in Supporting Information Fig. 1A.

### Statistics

Flow cytometry data were analyzed and enumerated using FlowJo (v10.2) and Graphpad Prism 6 (v6 Mac OS X). An unpaired, nonparametric Mann–Whitney *U* statistical test was used where applicable. Where more than 2 data sets are compared a one‐way ANOVA, nonparametric test was used. For each test, *p < 0.05, **p < 0.01, ***p < 0.001, and ****p < 0.0001. No bar present represents nonsignificant result.

## Conflict of interest

The authors declare no financial or commercial conflict of interest.

**Abbreviations**
Id2: inhibitor of DNA binding‐2
ILC: innate lymphoid cells
LTi: lymphoid tissue inducer
mLN: mesenteric lymph node
mTEC: medullary thymic epithelial cells
RANKL: receptor activator of nuclear factor kappa‐B ligand

## Supporting information

Peer review correspondence

Supporting Information

## References

[1] Artis, D. and Spits, H. , The biology of innate lymphoid cells. Nature. 2015 517: 293–301. 25592534 10.1038/nature14189

[2] Spits, H. , Artis, D. , Colonna, M. , Diefenbach, A. , Di Santo, J. P. , Eberl, G. , Koyasu, S. et al., Innate lymphoid cells—a proposal for uniform nomenclature. Nat. Rev. Immunol. 2013 13: 145–149. 23348417 10.1038/nri3365

[3] Eberl, G. and Littman, D. R. , The role of the nuclear hormone receptor RORgammat in the development of lymph nodes and Peyer's patches. Immunol. Rev. 2003 195: 81–90. 12969312 10.1034/j.1600-065x.2003.00074.x

[4] van de Pavert, S. A. and Mebius, R. E. , New insights into the development of lymphoid tissues. Nat. Rev. Immunol. 2010 10: 664–674. 20706277 10.1038/nri2832

[5] Rossi, S. W. , Kim, M. Y. , Leibbrandt, A. , Parnell, S. M. , Jenkinson, W. E. , Glanville, S. H. , McConnell, F. M. et al., RANK signals from CD4(+)3(‐) inducer cells regulate development of Aire‐expressing epithelial cells in the thymic medulla. J. Exp. Med. 2007 204: 1267–1272. 17502664 10.1084/jem.20062497 PMC2118623

[6] Scandella, E. , Bolinger, B. , Lattmann, E. , Miller, S. , Favre, S. , Littman, D. R. , Finke, D. et al., Restoration of lymphoid organ integrity through the interaction of lymphoid tissue‐inducer cells with stroma of the T cell zone. Nat. Immunol. 2008 9: 667–675. 18425132 10.1038/ni.1605

[7] Dudakov, J. A. , Hanash, A. M. , Jenq, R. R. , Young, L. F. , Ghosh, A. , Singer, N. V. , West, M. L. et al., Interleukin‐22 drives endogenous thymic regeneration in mice. Science. 2012 336: 91–95. 22383805 10.1126/science.1218004 PMC3616391

[8] Mackley, E. C. , Houston, S. , Marriott, C. L. , Halford, E. E. , Lucas, B. , Cerovic, V. , Filbey, K. J. et al., CCR7‐dependent trafficking of RORgamma(+) ILCs creates a unique microenvironment within mucosal draining lymph nodes. Nat. Commun. 2015 6: 5862. 25575242 10.1038/ncomms6862 PMC4354100

[9] Hepworth, M. R. , Monticelli, L. A. , Fung, T. C. , Ziegler, C. G. , Grunberg, S. , Sinha, R. , Mantegazza, A. R. et al., Innate lymphoid cells regulate CD4+ T‐cell responses to intestinal commensal bacteria. Nature. 2013 498: 113–117. 23698371 10.1038/nature12240 PMC3699860

[10] Gentek, R. , Munneke, J. M. , Helbig, C. , Blom, B. , Hazenberg, M. D. , Spits, H . and Amsen, D. , Modulation of signal strength switches notch from an inducer of T cells to an inducer of ILC2. Front. Immunol. 2013 4: 334. 24155745 10.3389/fimmu.2013.00334 PMC3804867

[11] Miyazaki, M. , Miyazaki, K. , Chen, K. , Jin, Y. , Turner, J. , Moore, A. J. , Saito, R. et al., The E‐Id protein axis specifies adaptive lymphoid cell identity and suppresses thymic innate lymphoid cell development. Immunity. 2017 46: 818–834.e4. 28514688 10.1016/j.immuni.2017.04.022 PMC5512722

[12] Wang, H. C. , Qian, L. , Zhao, Y. , Mengarelli, J. , Adrianto, I. , Montgomery, C. G. , Urban, J. F. et al., Downregulation of E protein activity augments an ILC2 differentiation program in the thymus. J. Immunol. 2017 198: 3149–3156. 28258196 10.4049/jimmunol.1602009 PMC5404348

[13] Wong, S. H. , Walker, J. A. , Jolin, H. E. , Drynan, L. F. , Hams, E. , Camelo, A. , Barlow, J. L. et al., Transcription factor RORalpha is critical for nuocyte development. Nat. Immunol. 2012 13: 229–236. 22267218 10.1038/ni.2208 PMC3343633

[14] Dutton, E. E. , Camelo, A. , Sleeman, M. , Herbst, R. , Carlesso, G. , Belz, G. T. and Withers, D.R. , Characterisation of innate lymphoid cell populations at different sites in mice with defective T cell immunity. Wellcome Open Res. 2017 2: 117. 29588921 10.12688/wellcomeopenres.13199.3 PMC5854988

[15] Constantinides, M. G. , McDonald, B. D. , Verhoef, P. A. and Bendelac, A. , A committed precursor to innate lymphoid cells. Nature. 2014 508: 397–401. 24509713 10.1038/nature13047 PMC4003507

[16] Klose, C. S. , Flach, M. , Mohle, L. , Rogell, L. , Hoyler, T. , Ebert, K. , Fabiunke, C. et al., Differentiation of type 1 ILCs from a common progenitor to all helper‐like innate lymphoid cell lineages. Cell. 2014 157: 340–356. 24725403 10.1016/j.cell.2014.03.030

[17] Ikawa, T. , Fujimoto, S. , Kawamoto, H. , Katsura, Y. and Yokota, Y. , Commitment to natural killer cells requires the helix‐loop‐helix inhibitor Id2. Proc. Natl. Acad. Sci. U S. A. 2001 98: 5164–5169. 11296270 10.1073/pnas.091537598 PMC33181

[18] Klose, C. S. , Kiss, E. A. , Schwierzeck, V. , Ebert, K. , Hoyler, T. , d'Hargues, Y. , Goppert, N. et al., A T‐bet gradient controls the fate and function of CCR6‐RORgammat+ innate lymphoid cells. Nature. 2013 494: 261–265. 23334414 10.1038/nature11813

[19] Roberts, N. A. , White, A. J. , Jenkinson, W. E. , Turchinovich, G. , Nakamura, K. , Withers, D. R. , McConnell, F. M. , et al., Rank signaling links the development of invariant gammadelta T cell progenitors and Aire(+) medullary epithelium. Immunity. 2012 36: 427–437. 22425250 10.1016/j.immuni.2012.01.016 PMC3368267

[20] Hikosaka, Y. , Nitta, T. , Ohigashi, I. , Yano, K. , Ishimaru, N. , Hayashi, Y. , Matsumoto, M. et al., The cytokine RANKL produced by positively selected thymocytes fosters medullary thymic epithelial cells that express autoimmune regulator. Immunity. 2008 29: 438–450. 18799150 10.1016/j.immuni.2008.06.018

[21] Akiyama, T. , Shimo, Y. , Yanai, H. , Qin, J. , Ohshima, D. , Maruyama, Y. , Asaumi, Y. , The tumor necrosis factor family receptors RANK and CD40 cooperatively establish the thymic medullary microenvironment and self‐tolerance. Immunity. 2008 29: 423–437. 18799149 10.1016/j.immuni.2008.06.015

[22] Abramson, J. and Anderson, G. , Thymic Epithelial Cells. Annu Rev Immunol. 2017 35: 85–118. 28226225 10.1146/annurev-immunol-051116-052320

[23] White, A. J. , Baik, S. , Parnell, S. M. , Holland, A. M. , Brombacher, F. , Jenkinson, W. E. and Anderson, G. , A type 2 cytokine axis for thymus emigration. J Exp Med. 2017 214: 2205–2216. 28694386 10.1084/jem.20170271 PMC5551576

[24] Oliphant, C. J. , Hwang, Y. Y. , Walker, J. A. , Salimi, M. , Wong, S. H. , Brewer, J. M. , Englezakis, A. et al., MHCII‐mediated dialog between group 2 innate lymphoid cells and CD4(+) T cells potentiates type 2 immunity and promotes parasitic helminth expulsion. Immunity. 2014 41: 283–295. 25088770 10.1016/j.immuni.2014.06.016 PMC4148706

[25] Rawlins, E. L. , Clark, C. P. , Xue, Y. and Hogan, B. L. , The Id2+ distal tip lung epithelium contains individual multipotent embryonic progenitor cells. Development. 2009 136: 3741–3745. 19855016 10.1242/dev.037317 PMC2766341

[26] Muzumdar, M. D. , Tasic, B. , Miyamichi, K. , Li, L. and Luo, L. , A global double‐fluorescent Cre reporter mouse. Genesis. 2007 45: 593–605. 17868096 10.1002/dvg.20335

[27] Luche, H. , Weber, O. , Nageswara Rao, T. , Blum, C. and Fehling, H. J. , Faithful activation of an extra‐bright red fluorescent protein in “knock‐in” Cre‐reporter mice ideally suited for lineage tracing studies. Eur J Immunol. 2007 37: 43–53. 17171761 10.1002/eji.200636745

[28] Jackson, J. T. , Hu, Y. , Liu, R. , Masson, F. , D'Amico, A. , Carotta, S. , Xin, A. et al., Id2 expression delineates differential checkpoints in the genetic program of CD8alpha+ and CD103+ dendritic cell lineages. EMBO J. 2011 30: 2690–2704. 21587207 10.1038/emboj.2011.163 PMC3155298

[29] Sun, Z. , Unutmaz, D. , Zou, Y. R. , Sunshine, M. J. , Pierani, A. , Brenner‐Morton, S. , Mebius, R. E. et al., Requirement for RORgamma in thymocyte survival and lymphoid organ development. Science. 2000 288: 2369–2373. 10875923 10.1126/science.288.5475.2369

[30] Eberl, G. and Littman, D. R. , Thymic origin of intestinal alphabeta T Cells revealed by fate mapping of RORgammat+ Cells. Science. 2004 305: 248–251. 15247480 10.1126/science.1096472

[31] Mombaerts, P. , Clarke, A. R. , Rudnicki, M. A. , Iacomini, J. , Itohara, S. , Lafaille, J. J. , Wang, L. et al., Mutations in T‐cell antigen receptor genes alpha and beta block thymocyte development at different stages. Nature. 1992 360: 225–231. 1359428 10.1038/360225a0

[32] Robinson, J. H. and Owen, J. J. , Pillars article: generation of T‐cell function in organ culture of fetal mouse thymus I. Mitogen responsiveness. 1975. J Immunol. 2008 181: 7437–7444. 19017929

