# Supplementary material for: Dynamic changes in intrathymic ILC populations during murine neonatal development
Source: Eur J Immunol. 2018 Jul 2;48(9):1481–91. doi: 10.1002/eji.201847511 (PMC6174991; doi:10.1002/eji.201847511)
Supplement: Supplementary file 2 — Supporting Information [file EJI-48-1481-s002.pdf]

European Journal of Immunology

Supporting Information

for

DOI 10.1002/eji.201847511

Rhys Jones, Emilie J. Cosway, Claire Willis, Andrea J. White, William E. Jenkinson, Hans J. Fehling, Graham Anderson and David R. Withers

Dynamic changes in intrathymic ILC populations during murine neonatal development

© 2018 The Authors. *European Journal of Immunology* published by WILEY-VCH Verlag GmbH & Co. KGaA, Weinheim.

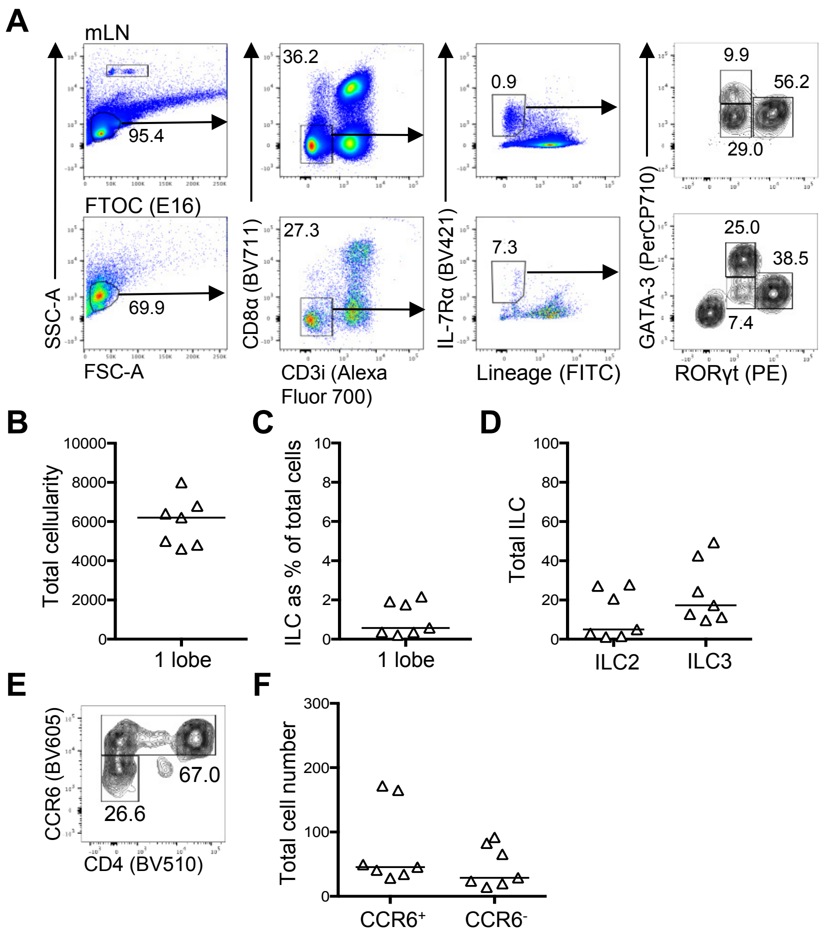

Supporting Information Figure 1: Identification of ILC populations in the embryonic thymus

Foetal thymic organ cultures (FTOC) using thymus lobes taken at embryonic day 16 were established to investigate ILC populations in the embryonic thymus. (A) Representative flow cytometry plots for the identification of ILC populations in the adult mLN (+ve control) and E16 FTOCs. Gating strategy in (A) used for identification of ILC subsets in embryonic, neonatal and adult tissue unless otherwise stated. (B) Total cellularity of 1 thymic lobe following 7 days in culture. (C) The percentage of ILC (CD8α$^-$CD3i$^-$IL-7Rα$^+$Lin$^-$ cells) as a proportion of total cellularity in 1 E16 FTOC. (D) Total number of ILC2 (GATA-3$^+$ ILC) and ILC3 (RORγt$^+$ ILC) in 1 E16 FTOC. (F) Representative flow cytometry plot (E) and total number (F) of LTi-like and non-LTi cells in the E16 thymus (pre-gated on RORγt$^+$ ILCs). Mann-Whitney U test was used for statistical analysis, n = 7 with data pooled from 2 independent experiments.

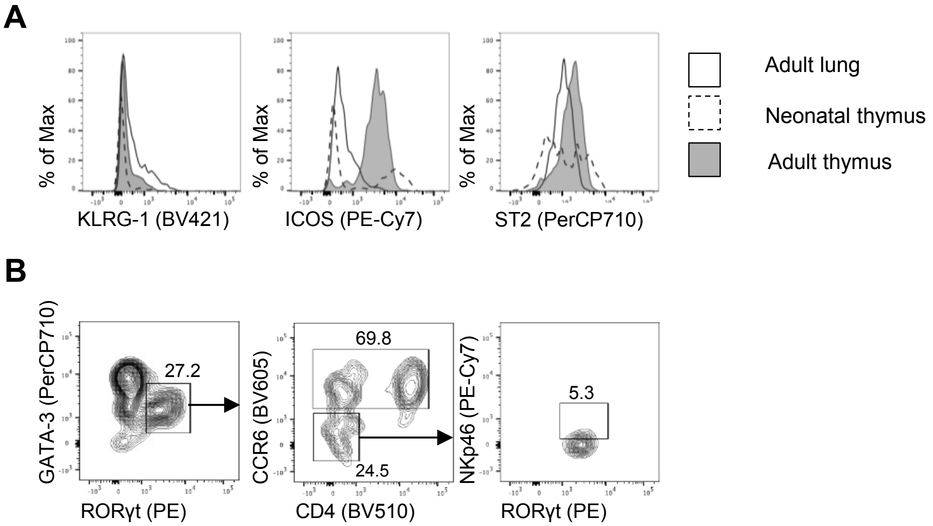

Supporting Information Figure 2: Characterisation of ILC2 and IC3 populations in the neonatal thymus

To assess the phenotype of ILC2 and ILC3 populations in the neonatal thymus the expression of ILC2 and ILC3 associated markers were compared. (A) Expression of KLRG-1, ICOS and ST2 by ILC2 isolated from day 7 neonatal thymus (dashed line), adult thymus (shaded histogram) and lung (solid line) tissue. (B) Expression of CCR6, CD4 and NKp46 on RORγt expressing ILC at 7 days post-birth. n=7 for (A) and (B) from 2 independent experiments.

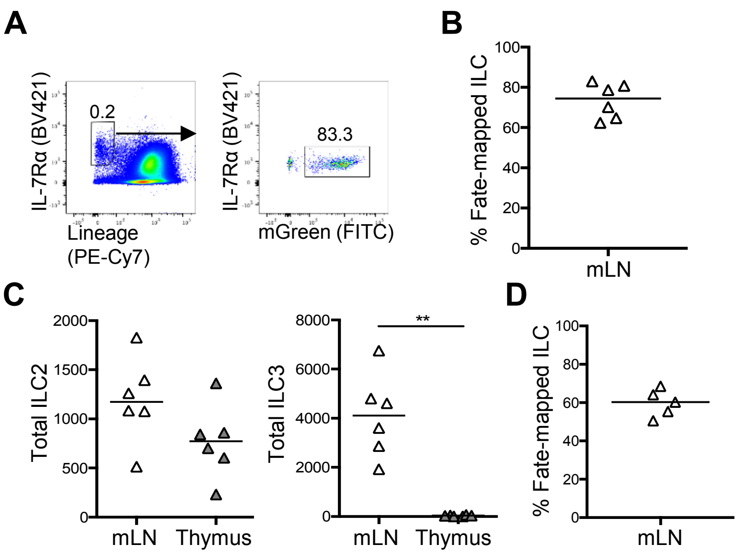

Supporting Information Figure 3. Fate mapping of ILC populations with administration of tamoxifen

Administration of tamoxifen in the diet was used as an alternative approach to enable prolonged tamoxifen exposure in Id2^{creERT2} x ROSA26$^{mT/mG}$ mice. (A) Representative flow cytometry plots of mGreen^{+} expression by ILC (IL-7Rα^{+}Lin^{-}CD8^{-} CD3i^{-}) in the mLN. (B) Median proportion of mGreen^{+} ILC in the mLN, used to calculate total numbers. (C) Total number of ILC2 (GATA-3^{+}) and ILC3 (RORγt^{+}) in the mLN and thymus. (D) Proportion of ILC fate-mapped to Id2 administered oral gavage in mLN. Mann-Whitney U test was used for statistical analysis where **=p<0.01. Bars show medians, n = 6 or n= 5 (in panel E) with data pooled from two independent experiments.

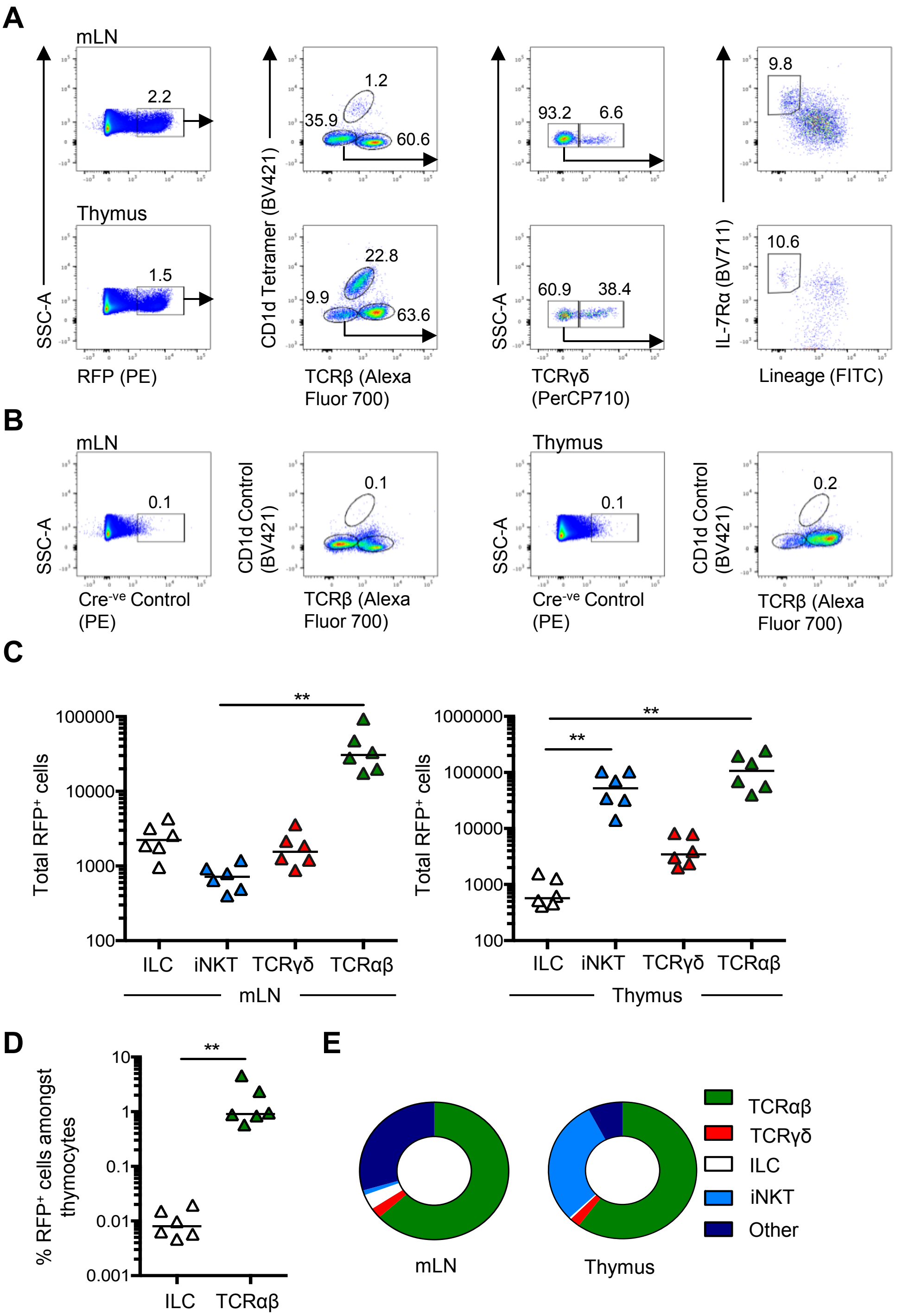

Supporting Information Figure 4. Fate mapping Id2 expression amongst thymocytes in Id2^{creERT2} x ROSA26^{tdRFP} mice

To assess which populations fate mapped for Id2 expression, Id2^{creERT2} x ROSA26^{tdRFP} mice were gavaged with tamoxifen for 5 consecutive days and then assessed 3 days later. (A) Representative flow cytometry plots showing identification of different immune cell populations in the mLN and thymus. (B) Controls supporting gating strategy for RFP$^+$ and CD1d tetramer$^+$ populations. (C) Total numbers of ILC, iNKT, $\gamma\delta$ TCR$^+$ and αβ TCR$^+$ cells amongst the RFP$^+$ population in the mLN and thymus cells. (D) The percentage of RFP$^+$ ILC and αβ TCR$^+$ cells amongst total thymocytes. (E) Graphical representation of the mean proportion of each RFP$^+$ population in the mLN and thymus. Mann-Whitney U test (comparing two samples) or a one-way ANOVA (comparing three or more samples) was used for statistical analysis where *= $p<0.05$, **=$p<0.01$. In all graphs the bar represents the median, n= 6. Data were pooled from 2 independent experiments.

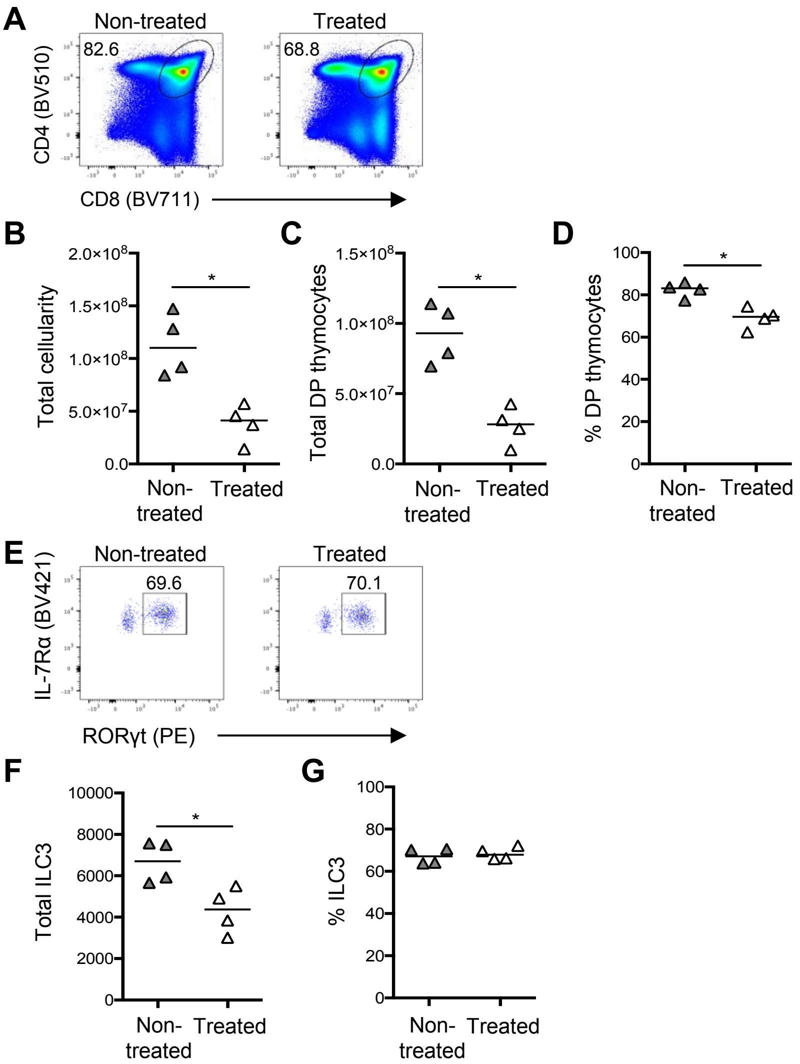

Supporting Information Figure 5. Oral gavage with tamoxifen results in loss of double-positive thymocytes

To assess the effect of acute tamoxifen administration on thymocyte development, WT mice were gavaged for 5 consecutive days with tamoxifen and then analysed 3 days later alongside untreated age-matched litter mates. (A) Representative flow cytometry plots showing expression of CD4 and CD8 amongst thymocytes. (B) Enumeration of total thymic cellularity in treated vs untreated mice. Number (C) and proportion (D) of double-positive (DP) thymocytes in treated versus untreated controls. (E) Identification of ILC3 in the mLN of treated and untreated mice. Number (F) and proportion (G) of ILC3 in the mLN of treated and untreated mice. Mann-Whitney U test was used for statistical analysis where *= $p<0.05$. In all graphs the bar represents the median, n= 4.

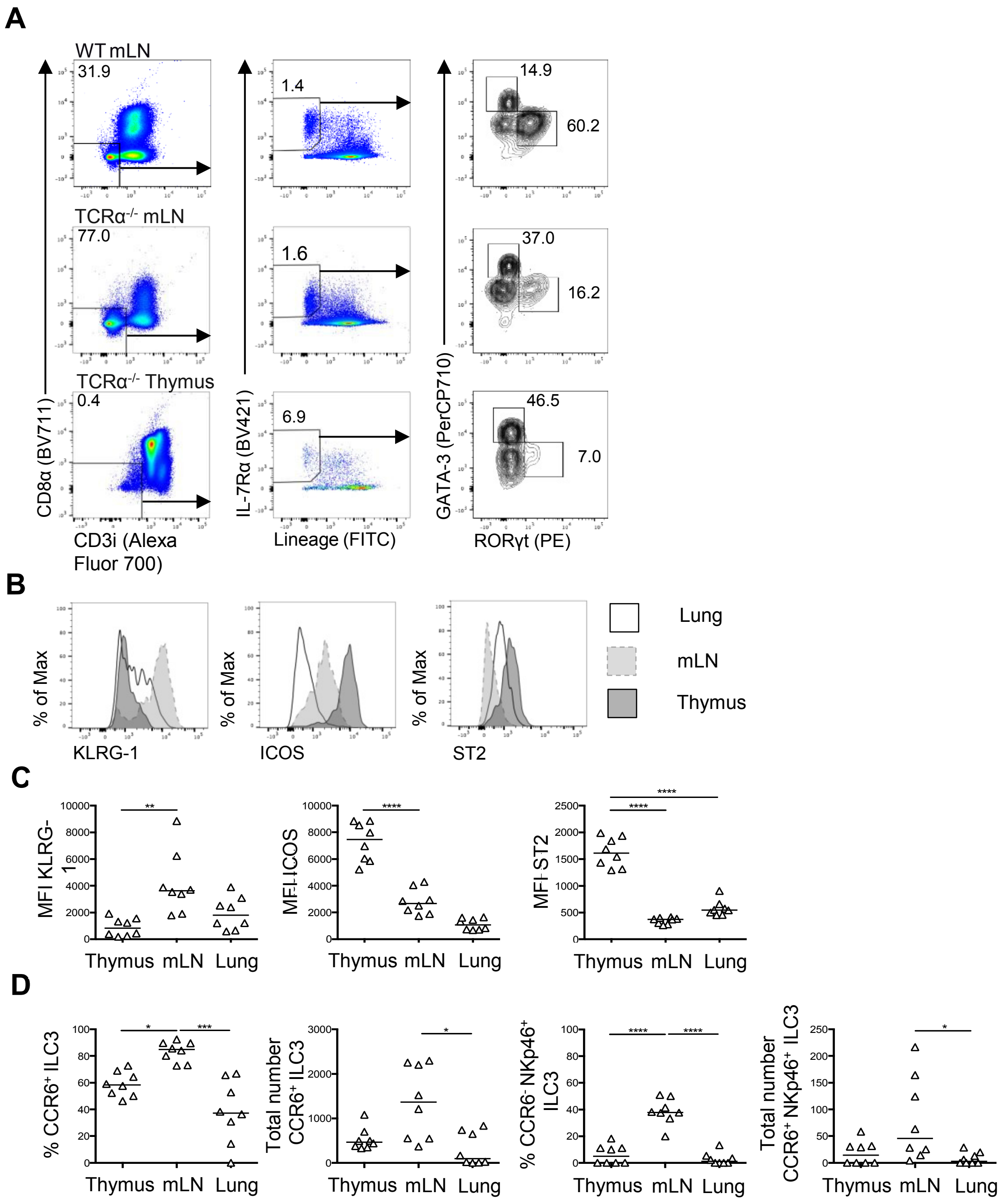

Supporting Information Figure 6. Identification of ILC populations in the TCRα$^{-/-}$ mice.

ILC populations within different tissues from TCRα$^{-/-}$ mice were assessed. (A) Representative flow cytometry plots for the identification of ILC (CD8α^{-}CD3i^{-}IL-7Rα^{+}Lin^{-}) in the mLN and thymus of TCRα$^{-/-}$ mice compared with mLN of WT mice, with ILC2 (GATA-3^{+}) and ILC3 (RORγt^{+}) assessed. (B) Expression of KLRG-1, ST2 and ICOS by ILC2 isolated from the thymus, mLN and lung of TCRα$^{-/-}$ mice. (C) MFI of KLRG-1, ST2 and ICOS by ILC2. (D) Percentage and total number of CCR6^{+} and CCR6^{-}NKp46^{+} ILC3 subsets in thymus, mLN and lung of TCRα$^{-/-}$ mice. One-way ANOVA was used for statistical analysis where *=p<0.05, **=p<0.01, ***=p<0.001 and ****=p<0.0001. Bars show median, n=8, with data pooled from 3 independent experiments.
