# Supplementary material for: Dynamic changes in intrathymic ILC populations during murine neonatal development
Source: Eur J Immunol. 2018 Jul 2;48(9):1481–91. doi: 10.1002/eji.201847511 (PMC6174991; doi:10.1002/eji.201847511)
Supplement: Supplementary file 1 — Peer review correspondence [file EJI-48-1481-s001.pdf]

European Journal of Immunology

Dynamic changes in intrathymic ILC populations during murine neonatal development

Rhys Jones, Emilie J. Cosway, Claire Willis, Andrea J. White, William E. Jenkinson, Hans J. Fehling, Graham Anderson, David R. Withers

Correspondence: Dr. David Withers, Institute of Immunology & Immunotherapy, College of Medical and Dental Sciences, University of Birmingham, B15 2TT, UK

Review Timeline:	Submission date:	24-Jan-2018
	First Editorial decision:	15-Feb-2018
	Revision received:	19-Apr-2018
	Accepted:	25-May-2018

Handling Executive Committee member: Prof. James Di Santo

Please note that the correspondence below does not include the standard editorial instructions regarding preparation and submission of revised manuscripts, only the scientific revisions requested and addressed.

First Editorial Decision
15-Feb-2018

Dear Dr. Withers,

Manuscript ID eji.201847511 entitled "Dynamic changes in intrathymic ILC populations during neonatal development" which you submitted to the European Journal of Immunology has been reviewed. The comments of the referees are included at the bottom of this letter.

A revised version of your manuscript that takes into account the comments of the referees will be reconsidered for publication. Should you disagree with any of the referees´ concerns, you should address this in your point-by-point response and provide solid scientific reasons for why you will not make the requested changes.

You should also pay close attention to the editorial comments included below. **In particular, please edit your figure legends to follow Journal standards as outlined in the editorial comments, please report the number of mice/samples per experiment. For flow cytometry data please show the full gating strategy. In the histograms/dot plots shown please report which fluorochromes were used and the scaling in the axis

(log/lin). Failure to do this will result in delays in the re-review process.**

Please note that submitting a revision of your manuscript does not guarantee eventual acceptance, and that your revision will be re-reviewed by the referees before a decision is rendered.

If the revision of the paper is expected to take more than three months, please inform the editorial office. Revisions taking longer than six months may be assessed by new referees to ensure the relevance and timeliness of the data.

Once again, thank you for submitting your manuscript to European Journal of Immunology and we look forward to receiving your revision.

Yours sincerely,
Marta Vuerich

On behalf of
Prof. James Di Santo

Dr. Marta Vuerich
Editorial Office
European Journal of Immunology
e-mail: ejied@wiley.com
www.eji-journal.eu

****

Reviewer: 1

Comments to the Author
This manuscript by Jones and colleagues provides the first detailed description of thymic ILC2 and ILC3, and the changes in relative and absolute abundance of these two immune cell populations over time.

The experiments described are carefully executed and the use of several in vivo models to identify ILC populations adds significantly to the strength of the study. In contrast to current thinking, the authors convincingly show that ILC2, rather than ILC3, are the dominant ILC population in the adult mouse thymus. This is an important message with immediate impact on ILC biology but especially on research addressing the role of ILCs in the context of thymic regeneration or damage responses.

European Journal of Immunology

Main points

Figure 4D and E. The constitutive production of IL13 by 100% of thymic ILC2 is not convincing. While the staining intensity is clearly higher than the FMO, it is also clearly lower than observed in stimulated cells or in non-stimulated ILC2 from any other organ. Based on those last two populations thymic ILC2 would seem negative for IL-13.
This claim needs to be substantiated by alternative methods to at least show production of IL-13 by non-stimulated thymic ILC2. Do these cells transcribe IL-13?

Figure 5. I have difficulty understanding the conclusion that the postnatal loss of ILC3 cannot be due to conversion to another ILC subtype. In figure 5B, 67% of mGreen ILC have lost Rorgt expression (while this is only 26% in mLN). Does this not show that more than half of the initial ILC3 have converted to a different lineage?

Page 10. ´Competition between ILC2 and ILC3 for a medullary niche in the thymus´This is not at all experimentally addressed in the manuscript and should be removed. Similarly, the statement to this effect that is included in the discussion should either be substantiated with arguments or removed.

Page 10. ´These data indicate that the expanding ILC2 population in the thymus do not take over the provision of RANKL to mTEC as ILC3 decline.´The experiments in figure 6 do not address RANKL levels in the presence of a declining ILC3 populations. RANKL levels should be compared between thymic ILC2 from WT mice and thymic ILC2 from Rorgt-/- mice to address whether RANKL expression on ILC2 increases in the absence of ILC3.

Minor points

It is not clear why the authors revert to the FTOC method to analyze fetal thymic ILC. Are insufficient numbers harvested from fetal thymic lobes to perform flow cytometry? If so, can the authors exclude effects of the 7 day culture on ILC numbers or distribution?

In general, the use of dot plots for flow cytometric data of small cell populations makes visual assessment difficult. I would advise to use density plots (as in fig 1H) for Ror/Gata; IL5/IL13 and RANKL stainings.

Reviewer: 2

European Journal of Immunology

Comments to the Author
In this study, Jones and colleagues report that the thymic ILC population includes both ILC3s and ILC2s. Thymic ILC3s have been shown previously to play an important role in the maturation of medullary epithelial cells through their expression of RANKL, while thymic ILC2s remain poorly characterized. Here, the authors show that the number of ILC2s increases 2 weeks after birth while the number of ILC3s drops significantly. ILC2s are located in the thymic medulla, do not derive from ´plastic´ILC3s, and appear to compete with ILC3s for their thymic niche.

This study adds significantly to our knowledge of different tissue-specific ILC subsets. However, a number of issues should be addressed and the impact of the manuscript could be significantly improved by providing additional information:

1. The authors characterize ILC2s and ILC3s by staining for Gata3 and Rorgt. Why have they not extended and completed their analysis of thymic ILC subset by including staining for T-bet and ILC1s?

2. In figure 1, it is shown that ILC2s increase expression of KLRG1 while they decrease expression of ICOS and ST2, comparing neonatal and adult thymi. These are important changes, in particular the loss of ST2, which must have functional consequences for the expression of cytokines by ILC2s. Could the authors test this and discuss the potential roles of type 2 effector cytokines in the neonatal thymus?

3. Two main figures are dedicated to tamoxifen-based fate mapping of Id2+ cells and a fine analysis of the fate mapped cells is reported in the supporting figure 3. Even though these data are interesting per see, they do not add to the information on ILCs gained by direct staining of the cells and by the Id2-GFP reporter mice, but rather confuse the reader.

4. In figure 4, the location of ILC2s in thymus is analyzed, for practical reasons, in TCRa-deficient mice. Even though I understand the rationale of the approach, the data obtained must be verified in wild-type mice, in order to avoid a potential ´KO´artefact.

5. In figure 5, RORgt-cre based fate mapping to assess whether thymic ILC2s are derived from ILC3s is the best way to test this hypothesis. However, the plot shown in figure 5B, and the few points found on the plot, makes it hard to derive any interpretation: more cells should be accumulated.

6. Figure 6 shows that RORgt-deficient mice have more ILC2s, prompting the authors to suggest that ILC2s and ILC3s compete for their thymic niche. Nevertheless, it is also possible that the modifications of the thymic stroma reported previously in RORgt-deficient mice impact on ILC2s. Therefore, thymic stromal cells from RORgt-deficient mice should be tested for their expression of cytokines known to promote

European Journal of Immunology

ILC2s, such as IL-25, IL-33 and TSLP.

First Revision – authors' response
19-Apr-2018

We thank the reviewers for their positive and supportive comments and have responded to the individual comments below. Changes to the manuscript text have been highlighted in yellow. To summarise the changes we have performed several further experiments requested by the reviewers including identification of ILC1 in the neonatal thymus, localisation of ILC2 in WT neonatal thymus and analysis of RANKL expression on ILC2 in WT versus Rorc-/- neonatal thymi. We have also amended our description of data as suggested by the reviewers. We hope that this revised manuscript meets with EJI publication standards.

Reviewer: 1
Comments to the Author
This manuscript by Jones and colleagues provides the first detailed description of thymic ILC2 and ILC3, and the changes in relative and absolute abundance of these two immune cell populations over time. The experiments described are carefully executed and the use of several in vivo models to identify ILC populations adds significantly to the strength of the study. In contrast to current thinking, the authors convincingly show that ILC2, rather than ILC3, are the dominant ILC population in the adult mouse thymus. This is an important message with immediate impact on ILC biology but especially on research addressing the role of ILCs in the context of thymic regeneration or damage responses.

Main points

1. Figure 4D and E. The constitutive production of IL13 by 100% of thymic ILC2 is not convincing. While the staining intensity is clearly higher than the FMO, it is also clearly lower than observed in stimulated cells or in non-stimulated ILC2 from any other organ. Based on those last two populations thymic ILC2 would seem negative for IL-13.
This claim needs to be substantiated by alternative methods to at least show production of IL-13 by non-stimulated thymic ILC2. Do these cells transcribe IL-13?

RESPONSE: We take the reviewers point. We were unable to source IL-13 reporter mice and felt the fairest way to present the ILC2 cytokine data was to omit the unstimulated data from Figure 4 and only describe what thymic ILC2 produced under ex vivo restimulation conditions. These data support that the cells identified as ILC2 in the thymus express the signature cytokines associated with ILC2 responses. We have changed the figure and the description of the data accordingly.

2. Figure 5. I have difficulty understanding the conclusion that the postnatal loss of ILC3 cannot be due to conversion to another ILC subtype. In figure 5B, 67% of mGreen ILC have lost Rorgt expression (while this is only 26% in mLN). Does this not show that more than half of the initial ILC3 have converted to a different lineage?

RESPONSE: We apologise for the confusion caused by this figure (a similar point was made by the second reviewer as well). Clearly our presentation and description of the data was inadequate. We have revised Figure 5 to try to make the following point more clear. Fate mapping of RORg cre expression enables the identification of exILC3. Given the surprising loss of ILC3 and the expansion of other ILC populations in the thymus after birth, we wanted to ask the question whether thymic ILC3 had converted to another ILC population. Whilst we considered this unlikely, we wanted to test this in vivo. As is hopefully clear in the revised Figure 5, only 20% of thymic ILC fate mapped (mGreen+) for RORgcre expression (versus 68% in the mLN). Therefore 80% of the ILC in the thymus have not expressed the RORg cre arguing that most thymic ILC2 are not derived from ILC3. Of the 20% mGreen+ ILC3 in the thymus, there is evidence that some of these are exILC3 given the lack of RORgt expression. We hope this point is now more clearly made.

3. Page 10. “Competition between ILC2 and ILC3 for a medullary niche in the thymus” This is not at all experimentally addressed in the manuscript and should be removed. Similarly, the statement to this effect that is included in the discussion should either be substantiated with arguments or removed.

RESPONSE: We agree with the reviewer and this is an over interpretation of our data given the potential caveats in the in vivo models used. We have omitted this description accordingly and changed the title of this section in the results. We simply describe the analysis of WT versus Rorc-/- thymi and conclude that this could reflect ILC intrinsic effects (i.e. competition) or simply the altered T cell development present in Rorc-/- mice. We hope this is now acceptable to the reviewer.

4. Page 10. “These data indicate that the expanding ILC2 population in the thymus do not take over the provision of RANKL to mTEC as ILC3 decline.” The experiments in figure 6 do not address RANKL levels in the presence of a declining ILC3 populations. RANKL levels should be compared between thymic ILC2 from WT mice and thymic ILC2 from Rorgt-/- mice to address whether RANKL expression on ILC2 increases in the absence of ILC3.

RESPONSE: We thank the Reviewer for this comment and we performed the experiment suggested. Interestingly, we observed significantly increased RANKL expression on ILC2 in the thymus of Rorc-/- mice. We have reported this surprising observation as we think this will be of interest although this experiment suffers from the issues the reviewer raises above and we simply state that ILC2 expression of RANKL can be altered in the presence of a disrupted thymic microenvironment. In the absence of clear models to test ILC2 function we have been unable to build further on this observation but this would be

interesting to explore in further studies.

Minor points

1. It is not clear why the authors revert to the FTOC method to analyze fetal thymic ILC. Are insufficient numbers harvested from fetal thymic lobes to perform flow cytometry? If so, can the authors exclude effects of the 7 day culture on ILC numbers or distribution?

RESPONSE: We have found it very difficult to isolate sufficient ILC from freshly isolated embryonic thymus and so have relied on the FTOCs. These have obvious caveats as the reviewer rightly points out and we have amended the text to make clear the limited conclusions that can be made from the FTOC data.

2. In general, the use of dot plots for flow cytometric data of small cell populations makes visual assessment difficult. I would advise to use density plots (as in fig 1H) for Ror/Gata; IL5/IL13 and RANKL stainings.

RESPONSE: We have revised the plots accordingly and show density plots now where populations are small in number

Reviewer: 2

Comments to the Author
In this study, Jones and colleagues report that the thymic ILC population includes both ILC3s and ILC2s. Thymic ILC3s have been shown previously to play an important role in the maturation of medullary epithelial cells through their expression of RANKL, while thymic ILC2s remain poorly characterized. Here, the authors show that the number of ILC2s increases 2 weeks after birth while the number of ILC3s drops significantly. ILC2s are located in the thymic medulla, do not derive from "plastic" ILC3s, and appear to compete with ILC3s for their thymic niche.

This study adds significantly to our knowledge of different tissue-specific ILC subsets. However, a number of issues should be addressed and the impact of the manuscript could be significantly improved by providing additional information:

1. The authors characterize ILC2s and ILC3s by staining for Gata3 and Rorgt. Why have they not extended and completed their analysis of thymic ILC subset by including staining for T-bet and ILC1s?

RESPONSE: We thank the reviewer for this comment and agree this data should be included. We have now included new data in an amended Figure 1 showing that ILC1 (based on Tbet expression) were detected in the neonatal thymus at D7 and D14 post birth.

European Journal of Immunology

2. In figure 1, it is shown that ILC2s increase expression of KLRG1 while they decrease expression of ICOS and ST2, comparing neonatal and adult thymi. These are important changes, in particular the loss of ST2, which must have functional consequences for the expression of cytokines by ILC2s. Could the authors test this and discuss the potential roles of type 2 effector cytokines in the neonatal thymus?

RESPONSE: We show comparisons of ILC2 surface marker expression in different ways and compare to both adult thymus and adult lung. In the revised Figure 1I (previously Fig 1G) the comparison is between neonatal thymus and adult lung (not thymus). This may have caused some of the concern noted by the reviewer. The point of this comparison was to compare thymic ILC to other better described ILC2 populations. A comparison using histograms of neonatal and adult thymic ILC2 has been taken out of Figure 1 (for space reasons) and now forms a revised Supporting Figure 2. Both neonatal and adult thymic ILC2 express very little KLRG-1, but substantial levels of ST2. Expression of ICOS is different, however, the functional relevance of this is unclear given conflicting publications on the importance of ICOS in ILC2 function. We think that the 'loss of ST2 expression' described by the reviewer reflects the difference between neonatal thymus and adult lung and we would make the point that both populations still express ST2.

We apologise for the confusion that our data presentation may have caused and we hope these data are now clearer.

We agree with the reviewer that we should provide some discussion of potential roles for type 2 effector cytokines in the neonatal thymus and have added this to the discussion, referring to the recent demonstration that type 2 cytokines from another innate population contribute to the control of thymocyte egress.

3. Two main figures are dedicated to tamoxifen-based fate mapping of Id2+ cells and a fine analysis of the fate mapped cells is reported in the supporting figure 3. Even though these data are interesting per see, they do not add to the information on ILCs gained by direct staining of the cells and by the Id2-GFP reporter mice, but rather confuse the reader.

RESPONSE: We thank the reviewer for this point but would politely disagree. We think that this detailed analysis using several different models adds weight and clarity to a situation that is confused by previous publications. We note that Reviewer 1 stated that: 'the use of several in vivo models to identify ILC populations adds significantly to the strength of the study'.

Our reasoning for including all of these different models is that (as with all models) there are caveats at each stage, but collectively all the data strongly supports our proposition. If we omit some of these data we think it would reduce the robustness of the analysis performed. Given the reviewer felt that these data caused confusion we have added minor amendments to the text to try to make clear why these data are important and what each set of data adds to the manuscript.

4. In figure 4, the location of ILC2s in thymus is analysed, for practical reasons, in TCRa-deficient mice. Even though I understand the rationale of the approach, the data obtained must be verified in wild-type mice, in order to avoid a potential "KO" artefact.

RESPONSE: We agree with the reviewer that this important analysis in WT thymi was missing and have now included data in a modified Figure 4 from D7 WT neonatal thymus where putative ILC2 were identified in a corresponding location (i.e. in the medulla), as described in the adult TCRα-/- thymus.

5. In figure 5, RORgt-cre based fate mapping to assess whether thymic ILC2s are derived from ILC3s is the best way to test this hypothesis. However, the plot shown in figure 5B, and the few points found on the plot, makes it hard to derive any interpretation: more cells should be accumulated.

RESPONSE: As also addressed above in response to Reviewer 1, we apologise that we failed to make the point clearly in this figure and have revised Figure 5 and its description to simply show that only 20% of thymic ILC fate map for RORγt expression. We hope that the reviewer thinks that the data is now more clearly presented. We have amended the text as well to help make the simple point regarding plasticity and thymic ILC3.

6. Figure 6 shows that RORgt-deficient mice have more ILC2s, prompting the authors to suggest that ILC2s and ILC3s compete for their thymic niche. Nevertheless, it is also possible that the modifications of the thymic stroma reported previously in RORgt-deficient mice impact on ILC2s. Therefore, thymic stromal cells from RORgt-deficient mice should be tested for their expression of cytokines known to promote ILC2s, such as IL-25, IL-33 and TSLP.

RESPONSE: As also suggested by Reviewer 1, we have amended our description of competition between thymic ILC2 and ILC3 in the manuscript to omit the text referred to in this point. It would be interesting to explore differences in thymic stroma in WT and Rorc-/- thymus but feel this is beyond the scope of this manuscript which is already substantial with 6 Main Figures and 6 Supporting Information Figures.

Second Editorial Decision

09-May-2018

Dear Dr. Withers,

It is a pleasure to provisionally accept your manuscript entitled "Dynamic changes in intrathymic ILC populations during neonatal development" for publication in the European Journal of Immunology. For final acceptance, please follow the instructions below and return the requested items as soon as possible as we cannot process your manuscript further until all items listed below are dealt with.

Please note that EJI articles are now published online a few days after final acceptance (see Accepted Articles: https://onlinelibrary.wiley.com/toc/15214141/0/ja). The files used for the Accepted Articles are the final files and information supplied by you in Manuscript Central. You should therefore check that all the information (including author names) is correct as changes will NOT be permitted until the proofs stage.

We look forward to hearing from you and thank you for submitting your manuscript to the European Journal of Immunology.

Yours sincerely,
Marta Vuerich

on behalf of
Prof. James Di Santo

Dr. Marta Vuerich
Editorial Office
European Journal of Immunology
e-mail: ejied@wiley.com
www.eji-journal.eu
